# Perspectives on the utility of moxidectin for the control of parasitic nematodes in the face of developing anthelmintic resistance

**DOI:** 10.1016/j.ijpddr.2019.06.002

**Published:** 2019-06-15

**Authors:** Roger K. Prichard, Timothy G. Geary

**Affiliations:** Institute of Parasitology, McGill University, Sainte Anne-de-Bellevue, Quebec, Canada, H9X3V9

**Keywords:** Moxidectin, Milbemycin, Ivermectin, Avermectin, Macrocyclic lactone resistance, Nematode

## Abstract

Macrocyclic lactone (ML) anthelmintics are the most important class of anthelmintics because of our high dependence on them for the control of nematode parasites and some ectoparasites in livestock, companion animals and in humans. However, resistance to MLs is of increasing concern. Resistance is commonplace throughout the world in nematode parasites of small ruminants and is of increasing concern in horses, cattle, dogs and other animals. It is suspected in *Onchocerca volvulus* in humans. In most animals, resistance first arose to the avermectins, such as ivermectin (IVM), and subsequently to moxidectin (MOX). Usually when parasite populations are ML-resistant, MOX is more effective than avermectins. MOX may have higher intrinsic potency against some parasites, especially filarial nematodes, than the avermectins. However, it clearly has a significantly different pharmacokinetic profile. It is highly distributed to lipid tissues, less likely to be removed by ABC efflux transporters, is poorly metabolized and has a long half-life. This results in effective concentrations persisting for longer in target hosts. It also has a high safety index. Limited data suggest that anthelmintic resistance may be overcome, at least temporarily, if a high concentration can be maintained at the site of the parasites for a prolonged period of time. Because of the properties of MOX, there are reasonable prospects that strains of parasites that are resistant to avermectins at currently recommended doses will be controlled by MOX if it can be administered at sufficiently high doses and in formulations that enhance its persistence in the host. This review examines the properties of MOX that support this contention and compares them with the properties of other MLs. The case for using MOX to better control ML-resistant parasites is summarised and some outstanding research questions are presented.

## Highlights

• Moxidectin (MOX) exhibits a long half-life and high potency.
• MOX shows low toxicity in the host and low ecotoxicity.
• MOX has properties suitable for high dose rate, long acting formulation.
• MOX can be effective against macrocyclic lactone (ML)-resistant parasites.
• Properties of MOX can be exploited to better control ML-resistant nematodes.

## Graphical abstract

**Figure:**
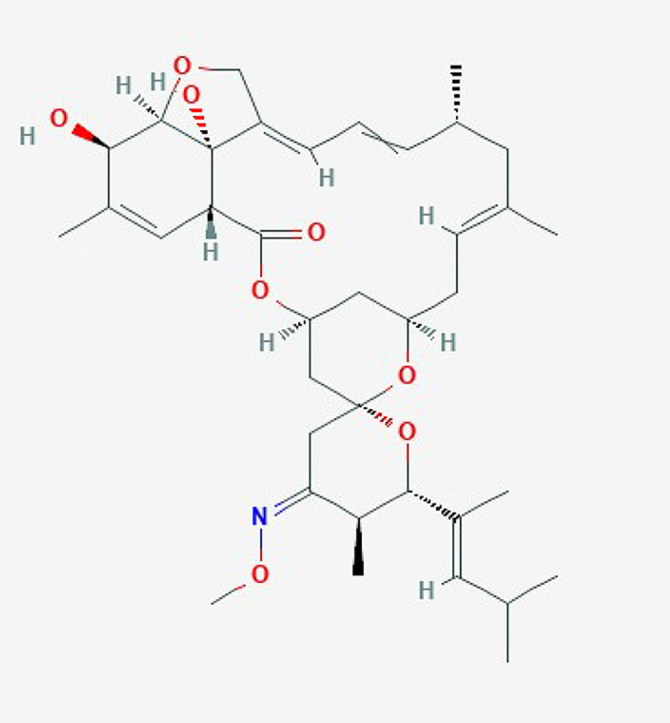

## 1 Introduction

Moxidectin (MOX) belongs to the macrocyclic lactone (ML) family of antiparasitic endectocides. The MLs are amongst the most widely used and successful anthelmintics. These hydrophobic, structurally related compounds are used in animals and humans, and also for pest control on crops. Most members of the ML family belong to the avermectin sub-family, whereas MOX is a milbemycin. Whilst there is considerable overlap between MOX and the commercially available avermectins in terms of endectocide spectrum, a number of important differences distinguish MOX from the avermectins (Prichard et al., 2012). In this review, we update the situation explored in the previous review, examine new information about these differences and present our perspectives on how some of the differences may be exploited to better maintain control of parasitic nematodes at a time when anthelmintic resistance (AR) is challenging parasite control.

### 1.1 Discovery of the macrocyclic lactones, including the milbemycins (milbemycin oxime and moxidectin) and the avermectins (ivermectin, abamectin, eprinomectin, doramectin and selamectin)

**Fig. 1:**
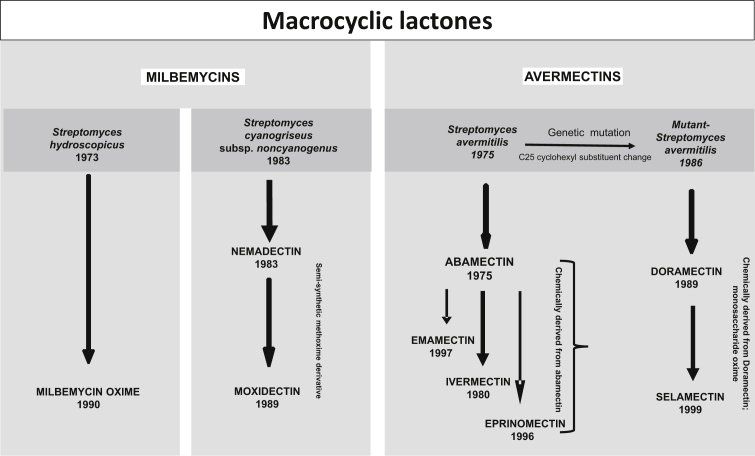
Historical development of macrocyclic lactone endectocides from different *Streptomyces* bacteria to therapeutic products.

Milbemycins were first isolated in 1967 from fermentation of a soil bacterium, *Streptomyces hygroscopes* and found to have insecticidal and acaricidal activity (Japan Patent application, 1973 No. 48-60127; Takiguchi et al., 1980). In 1972, the 16-membered ML structure of the active compound was elucidated and identified as milbemycin and from this discovery, the anthelmintic milbemycin oxime was derived (Takiguchi et al., 1983). Milbemycin oxime was approved for use as an anthelmintic in dogs in 1990 (FDA NADA 140-915 Interceptor, June 14, 1990). The relationship of the different commercial MLs to the bacteria that produce them is shown in Fig. 1.

**Fig. 2:**
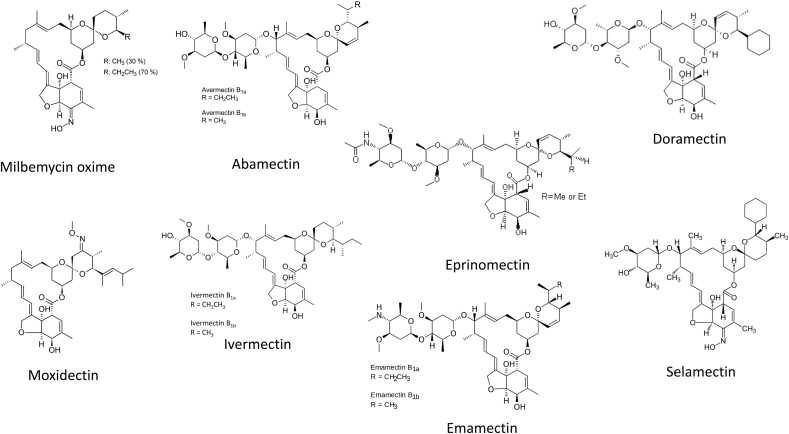
Structures of commercially available macrocyclic lactone parasiticides.

An active fermentation milbemycin product, nemadectin (F-29249α) (Doscher et al., 1989) was isolated from *Streptomyces cyaneogriseus* in 1983 and found to be a potent anthelmintic (Carter et al., 1988). MOX was later chemically derived from nemadectin by the addition of a methoxime moiety at C-23 (Fig. 2). It was highly efficacious against natural infections of cattle parasites (Ranjan et al., 1992).

The avermectins were derived from the soil bacterium *Streptomyces avermitilis* in 1975, and were selected on the basis of insecticidal and anthelmintic activity (Geary, 2005; Campbell, 2012). Avermectins are members of a group of pentacyclic 16-membered lactone compounds with endectocide activity (Campbell, 1989). The fermentation broth containing *S. avermitilis* was prepared in the laboratories of the Kitasato Institute, Japan and transmitted in 1974 to the laboratories of Merck & Co. Inc., USA, where the activities of abamectin against nematodes and many ectoparasites were discovered (Stapley and Woodruff, 1982). Abamectin is the mixture of avermectin B_1a_ (>90%) and avermectin B_1b_ (<10%) (Fig. 2). A-series compounds are methoxylated at the 5-position, whereas the B-series have an underivatized hydroxyl group at that position. The 1-subset compounds have an olefinic bond between C22 and C23; the 2-subset compounds possess a hydroxyl group at position 23 due to the hydration of the double bonds (Fig. 2). They are considered to have very similar biological activities and toxicological properties. Overall, avermectins are characterised among other MLs by the presence of a sugar substituent on the 13-position and of secondary butyl or isopropyl substituents in the 25-position.

Ivermectin (IVM), the most commonly used avermectin, is a chemically reduced 22,23-dihydro derivative of abamectin, and is a mixture of 22,23-dihydroavermectin B_1a_ (>90%) and 22,23-dihydroavermectin B_1b_ (<10%) (Fig. 2), differing from the components of abamectin by a single methylene group at the 26 position (Campbell, 1989).

Other commercially developed avermectins include emamectin, eprinomectin, doramectin and selamectin (Fig. 2). Emamectin was chemically derived from abamectin by substitution of an epi-amino-methyl (NHCH_3_) group for the hydroxyl (-OH) group at the 4″-position (i.e., in the terminal oleandrose sugar). Emamectin, like abamectin, is a mixture of two compounds, termed B_1a_ and B_1b_, which differ on the C-25 side-chain by one methylene (CH_2_) group. Eprinomectin is the amino-avermectin derived from avermectin B_1_ with a modified terminal oleandrose moiety called 4″-epiacetylamino-4″-deoxy-avermectin B_1_. Eprinomectin was identified, from IVM analogs synthesised by Merck, to have good efficacy, safety and a favourable milk residue profile (Shoop et al., 1996a,b). Doramectin is an avermectin prepared by mutational biosynthesis and it has a closer structural similarity to abamectin than to IVM (Goudie et al., 1993). There is a different substituent at the 25 position without the dihydro modification at the 22,23 position. It differs from IVM by having a cyclohexyl group in the C25 position of the avermectin ring. Its chemical name is 25-cyclohexyl-5-O-demethyl-25-de(1-methylporpyl) avermectin A_1_. Selamectin is a semisynthetic monosaccharide oxime derivative of doramectin. This drug was selected for efficacy against heartworms and for providing utility against fleas at a dose that is safe for use in dogs and cats (Banks et al., 2000). In terms of chemical structure and because of its monosaccharide, it is an interesting intermediate between the disaccharide avermectins and milbemycins.

Abamectin and IVM were the first MLs with very broad-spectrum activity developed for commercial use in animals in the early 1980s. MOX, while not the first milbemycin to be commercially developed, also showed very broad-spectrum activity. The milbemycins and avermectins, referred to as endectocides because of their activity against endoparasites and ectoparasites, have been important in the agricultural chemical industry because of their extremely high activity against arthropod and nematode pests, low toxicity to mammals, and unique mode of action. The milbemycins and avermectins have a common pharmacophore: the 16-member macrocyclic lactone ring fused with both benzofuran and spiroketal functions in a three-dimensional arrangement, which is recognised by specific chloride ion channel receptors. The high affinity binding of IVM, MOX and other MLs to these receptors is responsible for the mechanism of action of the ML class of drugs. IVM and MOX in particular have revolutionized parasite control in production animals, heartworm disease prevention in companion animals and antifilarial chemotherapy in humans.

However, the structural differences, related to the presence or absence of various substituents, affect the pharmacokinetics, pharmacodynamics and toxicity of MOX compared with the avermectins, and indeed compared with milbemycin oxime. Because MLs have systemic actions and must cross the tissues of the host organism before reaching the target parasite, drug disposition in the host, e.g., concentration and half-life, are important components of drug efficacy and utility. Thus, any factor that modulates the amount of active drug that reaches the target and the duration of its effects is of major importance. Physicochemical properties are important in modulating the rate of drug exchange between the tissues and the blood stream.

## 2 Physico-chemical properties of moxidectin

MOX shares a common 16-membered macrocyclic structure with other MLs, fused to benzofuran and spiroketal functions. As noted above, it differs from nemadectin by the addition of a methoxime moiety at C-23 (Fig. 2). Unlike the avermectins, milbemycins are non-glycosylated and differ from avermectin aglycones by being protonated at the C-13 position, where avermectin aglycones are hydroxylated. They also have an aliphatic group at the 25-position. Milbemycin derivatives differ in substituents in the 5-and 25-positions; the substituted olefinic side chain at the 25-position and the methoxime moiety at the 23-position are specific to MOX, not present in other commercial milbemycins or avermectins. MOX is more lipophilic than other macrocyclic lactone endectocides, with a logP of 5.4, compared for example with IVM, which has a logP of 4.3. The extent to which the lipophilicity of MOX per se accounts for its pharmacological advantages, as opposed to its specific pattern of substituents, remains to be experimentally determined.

## 3 Current uses, including potency and efficacy, of moxidectin, milbemycin oxime and the avermectins in veterinary medicine

The activities of MOX and the avermectins against nematodes and ectoparasites have been comprehensively reviewed in Prichard et al. (2012), and here only new information and significant differences between MOX, milbemycin oxime and the avermectins will be highlighted. It should be noted that milbemycin oxime and selamectin are only used in dogs and cats. Besides their use in dogs and cats, MOX and IVM are used in many other animal species, including cattle, sheep, and horses. IVM and doramectin, but not MOX, are used in pigs. Doramectin and eprinomectin are used in cattle and sheep, with eprinomectin having a zero-day milk withholding period in milking ruminants.

The preferred route of administration for different MLs depends on the target species. In cattle, pour-on formulations are popular because of ease of administration. However, injectable formulations are also available and are the route of administration for long-acting formulations such as Cydectin®LA and Longrange^®^ (see discussion in Section 4). For sheep, horses and pigs, the oral route of administration is usually preferred.

For heartworm and gastrointestinal (GI) worm control in dogs, a chewable oral formulation is usually used, with the exception of injectable long-acting ProHeart^®^6 and ProHeart^®^ SR-12 formulations of MOX, and topical formulations of MOX (Advantage Multi^®^, Advocate^®^, Coraxis^®^ (which is not combined with an ectoparasiticide)) and selamectin (Revolution^®^). The spectra and use of the different MLs were reviewed in Prichard et al. (2012). However, since then, the spectrum of MOX (2.2 mg/kg) in Advantage Multi^®^/Advocate^®^ has been extended to include activity against *Dirofilaria immitis* microfilariae (Bowman et al., 2015). Topical MOX is the only heartworm product registered as a microfilaricide. In addition, a number of new oral formulations of milbemycin oxime (at a standard minimum dose of 0.5 mg/kg) in combination with an ectoparasiticide have been registered to give broad-spectrum nematode and ectoparasite activity in dogs.

## 4 Lessons from registration of moxidectin and ivermectin for use in humans

Soon after its efficacy and safety against parasitic nematodes in animals were established, IVM was tested for the control of onchocerciasis in humans (Aziz et al., 1982a, 1982b). In lightly infected individuals residing in Senegal, IVM was highly efficacious against the microfilarial stage of *Onchocerca volvulus*, the filarial nematode that causes River Blindness in people. The previously available drug for treatment of onchocerciasis, diethylcarbamazine, causes a syndrome of severe adverse events known as the Mazzotti reaction. Remarkably, IVM treatment of *O. volvulus*-infected people caused minimal if any clinical reaction and was highly efficacious at removing microfilariae from the skin and eyes (Greene et al., 1989; Brown, 2002) and sterilizing adult worms for three to six months. These combined effects markedly reduce morbidity and transmission. In 1985, IVM was approved for use in humans at a dose of 150 μg/kg for onchocerciasis (Lindley, 1987), as Mectizan^®^, and its maker, Merck and Company, agreed to donate it for human use against onchocerciasis for as long as necessary. It became the main treatment modality for onchocerciasis by the Onchocerciasis Control Programme (OCP) in West Africa since 1987, and subsequently for the African Programme for Onchocerciasis Control (APOC), as well as for various national programs, mostly using annual treatment of communities in endemic areas. It was also the main tool for the Onchocerciasis Elimination Program for the Americas (OEPA), where twice per year treatments were employed. Subsequently, Mectizan^®^ treatment was extended for lymphatic filariasis in sub-Saharan Africa where diethylcarbamazine cannot be used due to the possibility of the Mazzotti reaction when concurrent *O. volvulus* infection may occur.

To date, well over a billion doses of Mectizan^®^ have been administered safely to humans. The exception has been a small number of individuals with concurrent high levels of *Loa loa* microfilaraemia (>30,000 microfilariae/ml blood). In such individuals, there is a significant risk of severe adverse events (SAE) to IVM that can lead to coma and death within a short period of time. These IVM-associated SAEs appear to be linked to both the concurrent *L. loa* infections and to the microfilaricidal action of IVM on *L. loa* microfilariae, possibly the result of the death of large numbers of *L. loa* microfilariae in the central nervous system (Boussinesq et al., 2003).

The use of IVM to reduce morbidity and transmission of onchocerciasis has in many endemic areas markedly reduced the incidence and prevalence of the disease, and in many formerly endemic countries of Central and South America it has eliminated infection. However, in parts of Africa, while the incidence of disease has markedly diminished, transmission of infection can persist despite more than 30 years of annual and more recently biannual treatment in some areas. Situations of ‘sub-optimal’ responses to IVM treatment have been reported in Ghana, Cameroon and Sudan (Ali et al., 2002; Osei-Atweneboana et al., 2007; Pion et al., 2013) and the possibility of resistance to IVM in *O. volvulus* has been recognized (Osei-Atweneboana et al., 2011).

A number of *in vivo* studies demonstrated the potent activity of MOX on microfilariae (Tagboto and Townson, 1996) and anti-reproductive effects on adult *Onchocerca* spp. (Trees et al., 2000). The greater potency of MOX against the filarial nematode *D. immitis* compared with other MLs, such as IVM, has been recognized for some time (McCall et al., 1992; McTier et al., 1992; Blagburn et al., 2011) and suggests that this may be generally true for filariids.

MOX is safer than IVM in some breeds of dogs, such as collies, which often have a defective *mdr1* gene (see Prichard et al., 2012 for review), a finding which suggested that MOX could be at least as safe as IVM and at least as efficacious for treatment of onchocerciasis in humans. In 1999, Wyeth Research initiated a collaboration with the World Health Organization to evaluate MOX for the treatment of onchocerciasis in humans. At least eight clinical studies on the safety and use of MOX for this indication have been completed (see e.g., Opoku et al., 2018). MOX showed superior effects in suppressing microfilarial counts up to 18 months compared with IVM, which has antifertility effects typically lasting 3–6 months (or less in sub-optimal responders). MOX was registered for use in humans for the treatment of onchocerciasis by the US Food and Drug Administration on June 13, 2018 (https://www.prnewswire.com/news-releases/us-fda-approves-moxidectin-for-the-treatment-of-river-blindness-300666114.html). The safety and persistent efficacy of MOX onchocerciasis patients support the hypothesis that this drug may provide improved control of veterinary filarial parasites in the face of developing ML resistance in canine heartworm infections.

Besides the use of MOX for control of filarial infections in humans, it shows promise for improving control of scabies in humans caused by *Sarcoptes scabiei* (Bernigaud et al., 2016; Mounsey et al., 2016), strongyloidiasis caused by *Strongyloides stercoralis* (Barda et al., 2017) and other GI nematode infections (Maheu-Giroux and Joseph, 2018). The increased use of MOX for treating human infections will be greatly facilitated by its recent registration for treatment of onchocerciasis and its long record as a safe pharmaceutical in animal health.

## 5 Mode of action and pharmacodynamics of moxidectin compared to other macrocyclic lactones

MOX, like other ML endectocides, acts by binding at sub-nanomolar concentrations to glutamate-gated chloride channels (GluCls) in a pseudo-irreversible manner, leading to flaccid paralysis of neuromuscular systems in nematodes and arthropods. GluCls are members of the cys-loop ligand-gated ion channel family, found in nematodes and some arthropods, but not in vertebrates, making them ideal drug targets for selective activity against parasites in mammals. The pharmacodynamics of MOX and the avermectins has been previously reviewed (Prichard et al., 2012; Wolstenholme, 2012; Kotze and Prichard, 2016). GluCls are encoded by the *avr-14, avr-15, glc-1, -2, -3, -4, -5* and *-6* genes (Glendinning et al., 2011), which are highly conserved in free-living and parasitic nematodes (Wolstenholme and Rogers, 2005; Li et al., 2014). They are widely expressed in the nematode nervous system (Wolstenholme and Rogers, 2005) and are involved in muscular contraction, locomotion, reproduction, feeding, excretory pore secretion, and mediation of sensory input (Wolstenholme, 2012). In the filarial nematode *Brugia malayi*, the *avr-14* gene is strongly expressed in early embryos, the wall of the uterus, and in the reproductive apparatus of adult males (Li et al., 2014). In microfilaria of *B. malayi, avr-14* is the only GluCl expressed, and is localized in the excretory pore (Moreno et al., 2010). It is important to extend such studies to other filariae, such as *D. immitis*, to determine whether this is characteristic of filariae in general.

MOX binds with high affinity and essentially irreversibly to the transmembrane domain of a GluCl from *Haemonchus contortus* and increases influx of Cl^−^ ions, resulting in hyperpolarization and muscle paralysis. In contrast to the effect of glutamate on potentiating IVM binding, for which 10 μM glutamate resulted in a 7-fold increase in IVM affinity, the same concentration of glutamate caused only a 1.5-fold increase in MOX affinity, suggesting that while both MOX and IVM bind to the same receptor site, the interaction with the receptor differs between MOX and IVM in the presence of the natural ligand (Forrester et al., 2002). In another study, the effects of IVM and MOX on opening homomeric *Cooperia oncophora* GluClα3 (a homolog of Cel-avr-14) expressed in *Xenopus* oocytes were compared. After wash-out of glutamate, IVM had an EC_50_ of 0.5 μM, while MOX had an EC_50_ of 0.2 μM. An allele of this receptor isolated from IVM-resistant *C. oncophora*, containing the L256F single nucleotide polymorphism (SNP), was 2.5 times more sensitive to MOX than to IVM (Njue et al., 2004). These authors concluded that MOX was a more potent agonist than IVM for GluCls expressed in *Xenopus* oocytes. While these differences are not quantitatively large and differ depending on the particular study, they lead to the conclusion that MOX and IVM do not interact with GluCls in an identical manner. Still unknown are possible differences in affinity of MLs for the several types of GluCls expressed in a nematode species, and whether affinities for them also differ among homologous channels in different species.

Structural studies support the conclusion that MOX and IVM differ at least to some degree in receptor binding. Based on the crystal structure of *C. elegans* GLC-1 in the presence of IVM, Hibbs and Gouaux (2011) proposed a model for the IVM binding sites and atomic interactions with receptor amino acids. The chemical functions of IVM that appear to bind to amino acids of the receptor via van der Waals and H-bond interactions were determined. The structural differences between IVM (as a representative avermectin) and MOX (absence of the disaccharide moiety (or -OH) on C-13 of the macrocycle, a methoxime moiety at C-23 and an olefinic side chain at C-25) suggest that the interaction of MOX differs from that of the avermectins. Four of the interaction sites involved with IVM binding to the GluCl were retained for MOX binding, but three of the proposed IVM interaction sites are not present or are blocked when MOX is fitted to the same receptor (Prichard et al., 2012). The methoxime on the spirokeletal ring of MOX may prevent H-binding to a M3 loop and may cause some molecular displacement of this loop, or another interaction site, while the absence of a disaccharide substituent should result in MOX lacking two van der Waal binding sites (to the M2-M3 loops) in the nematode GluCl (Prichard et al., 2012). This suggests that MOX may interact differently with GluCls than IVM.

Physiological studies on the effects of IVM and MOX on pharyngeal pumping and motility in adult *C. elegans* and on larval development (Ardelli et al., 2009) support the interpretation of the structural studies; these drugs do not exert the same effects at similar concentrations on this nematode. For example, 5 nM IVM paralyzed pharyngeal pumping, but a similar paralysis required 80 nM MOX. For worm motility, IVM caused an initial activation of *C. elegans*, an effect not seen after MOX exposure. Larval development was very sensitive to IVM, being inhibited at 0.6 nM, whereas a 64-fold higher concentration of MOX was required to inhibit development. In experiments with IVM-sensitive adult *H. contortus*, pharyngeal pumping was also found to be significantly more sensitive to IVM in the presence of glutamate compared to MOX; in contrast, in an IVM-resistant strain of *H. contortus*, IVM was significantly less potent on pharyngeal pumping, while responses to MOX did not change (Paiement et al., 1999). Thus, MOX may act on the same receptors as other MLs, but there appear to be significant differences in the characteristics of MOX and avermectin interactions, presumably due to the unique chemical properties and structural characteristics of MOX compared with avermectins and other milbemycins.

## 6 Pharmacokinetics of moxidectin compared with other macrocyclic lactones

The concentration and length of residence time of a ML in a target host tissue is an important determinant of the efficacy of the drug against parasites in, or associated with, that tissue. Many studies that evaluated and compared the pharmacokinetics of different MLs were previously reviewed (Lespine et al., 2012; Prichard et al., 2012). Pertinent differences between MOX and most other MLs include that MOX is characterized by a much larger volume of distribution, a remarkably long mean residence time in host tissues, high plasma and lipid concentrations and a relatively large area under the plasma concentration versus time curve (AUC). In cattle, MOX has a larger volume of distribution and faster (plasma) clearance than IVM (Lanusse et al., 1997; Bousquet-Melou et al., 2004), presumably due to a more rapid partition into adipose tissue. The mean residence time is longer for MOX than for IVM (24 vs. 7 days, respectively) after IV injection (Bousquet-Melou et al., 2004). The concentration of MOX in fat 28 days after treatment was 90-fold higher than in plasma and the half-life of MOX was 14 days vs. 7 days for IVM (Zulalian et al., 1994). Eprinomectin presents a pharmacokinetic profile similar to that of IVM in cattle, except that it does not partition into milk (Lespine et al., 2003; Baoliang et al., 2006). In dogs, MOX and IVM pharmacokinetics differ after oral administration, with a longer elimination half-life and larger volume of distribution for MOX (Al-Azzam et al., 2007).

MLs can be formulated in different ways, which affects absorption from the site of administration in a given species. However, differences in the elimination phase, reflected in the half-life of elimination, are primarily related to differences in ML lipophilicity and efflux potential via ABC transporters. As a consequence, the higher lipophilicity of MOX compared with IVM or eprinomectin (log*P*_epm_ = 4.0) favours the retention of MOX in fatty tissues.

As discussed below (Section 7), differences in the interaction of MOX and avermectins with ABC transporters likely also play a role in the unique pharmacokinetic attributes of MOX that contribute to its sustained efficacy. This lipophilicity of MOX permits convenient administration by different routes, according to the species of animal being treated, and provides high and sustained blood and tissue levels. For example, MOX is typically administered to sheep as an oral drench at 200 μg/kg and produces a peak concentration on day 1 and a mean residence time of 12.5 days (Alvinerie et al., 1998), although in some countries a long-acting Cydectin®LA subcutaneous injectable formulation (1 mg/kg) is available for this species and provides a much longer duration of exposure. MOX can be administered to cattle as a pour-on (500 μg/kg), an injectable (200 μg/kg), or as a long-acting injectable formulation, Cydectin®LA (1 mg/kg), which can provide up to 120 days of protection against some parasitic nematodes. MOX can be administered to dogs at an oral dose of only 3 μg/kg to provide >30 days protection against heartworm infection, as a subcutaneous injection in the ProHeart^®^6 or ProHeart®SR12 formulations, which provide protection against heartworm for 6 and 12 months, respectively, and remove a number of GI worms present at the time of treatment. MOX is also provided as a spot-on formulation (dogs and cats), Coraxis^®^ (2.2 mg MOX/kg) or Advantage Multi^®^/Advocate^®^ (2.2 mg MOX/kg combined with the ectoparasiticide imidacloprid at 8.8 mg/kg), which provides heartworm protection for > 30 days, *D. immitis* microfilaricidal efficacy, removal of other parasitic nematode, and in the case of the combination products, flea control (mainly due to imidacloprid). In this spot-on formulation, after two monthly treatments in dogs, MOX plasma concentrations were maintained in excess of the peak concentration measured after a single dose, reflecting accumulation, and plasma levels of MOX could be detected in dogs treated with four monthly doses for more than 22 weeks after the last treatment (Bowman et al., 2016). In cats, Advantage Multi^®^ treatment also achieves a long half-life of 28 days and treatment each month can cause additive increases in blood levels (Little et al., 2015), potentially boosting efficacy.

### 6.1 Elimination in milk

The mammary gland epithelium acts as a lipid barrier, and many lipophilic drugs readily diffuse from plasma into milk. The high lipophilicity of the MLs assists in their partitioning into milk. Milk/plasma concentration ratios close to unity have been reported for IVM in many species (Prichard et al., 2012). Consistent with the higher lipophilicity of MOX, its partitioning into milk is higher than for IVM. On the other hand, eprinomectin is less lipophilic than MOX or IVM and is a better substrate for mammalian ABC transporters, resulting in a low milk to plasma concentration in cattle of 0.2 (Shoop et al., 1996b), which is advantageous in terms of milk residues.

### 6.2 Biotransformation

Host and parasite enzymes can metabolise MLs and contribute to drug elimination and disposition. IVM is primarily excreted as parent compound. However, it is partially metabolized to 24-hydroxy-ivermectin, O-desmethyl-ivermectin and 3-O-desmethyl metabolites in different mammals (Prichard et al., 2012). Cytochrome P4503A4 is an important enzyme for IVM metabolism in humans and rats (and presumably other mammals) (Zeng et al., 1998). MOX is metabolized to C29-30- and C14-mono-hydroxy-methyl derivatives as the main products (Zulalian et al., 1994). In cattle, the fractions metabolized were around 8% for IVM and 13% for MOX. The clearance reflects the capacity of the organism to eliminate a drug from plasma. The higher clearance of MOX calculated in cattle after IV administration (Lanusse et al., 1997; Bousquet-Melou et al., 2004) could be due to higher metabolism or larger volume of distribution compared with IVM. However, metabolism is considered to contribute relatively little to ML elimination compared with excretion of parent compound.

### 6.3 Pharmacokinetics of formulations that enable long-acting injection of macrocyclic lactones

Because of their low toxicity minimizing concerns over duration of exposure, formulations of MOX and IVM were developed which provide tissue depots and very prolonged durations of antiparasitic activity. Perhaps the most remarkable long-acting formulations are for MOX in ProHeart^®^6 and ProHeart®SR-12. In these formulations, MOX is contained in microspheres that ensure its sustained slow release, preventing heartworm disease for 6 and 12 months, respectively, after a single injection. The long duration of action of MOX in these formulations is facilitated by its high lipophilicity, which complements the slow release of very small amounts of the active ingredient from the microspheres, resulting in remarkable effectiveness against developing *D. immitis* larvae. The remarkable potency of the drug enables extremely low plasma concentrations to block development to the pathogenic adult stage.

Other long-acting formulation of MOX are Cydectin®LA for cattle, which can provide up to 120 days of protection against some parasitic nematodes, and Cydectin®LA for sheep, which can provide control of some nematode species for 44–111 days. For both cattle and sheep, these long-acting injectable MOX formulations are administered subcutaneously at a dose of 1 mg/kg. In cattle, peak plasma concentrations occur at day 3–4, but by 120 days after administration, a plasma concentration of 1.9 ng/ml was still present (Dupuy et al., 2007). These products demonstrate the remarkable and indeed unique flexibility of formulation, potency and therapeutic longevity of MOX.

Another example of a long acting depot ML injectable is Longrange^®^ in cattle. This formulation contains eprinomectin in a poly-lactide-co-glycolic-acid polymer carrier that degrades over time at the subcutaneous site of injection, providing up to 150 days of therapeutic activity against some species of nematodes. It has a particularly interesting pharmacokinetic profile with an initial high peak of plasma eprinomectin around day 3, followed by an extended period of low-level eprinomectin release, and then a second broad peak at around 93 day as the polymer carrier degrades, releasing the residual eprinomectin. This prolonged release profile reduces production losses due to nematode parasites over the typical summer grazing season in temperate regions of the world.

## 7 Interactions with multidrug ABC transporters

ABC transporters can have major effects on the uptake and excretion of MLs in mammalian hosts and in nematode parasites. The interactions of different MLs with mammalian and parasite ABC transporters have been extensively reviewed (Lespine et al., 2008, 2012; Prichard et al., 2012). However, additional information pertinent to safety, pharmacokinetics and efficacy has become available since these publications and is reviewed briefly, as it is relevant for efforts to control parasitic nematodes in the face of developing resistance.

### 7.1 Mammalian ABC transporters

IVM was the first ML shown to be a ligand for mammalian P-glycoprotein (ABCB1) (Didier and Loor, 1996; Pouliot et al., 1997). IVM inhibits the transport of a number of P-glycoprotein substrates, as it is transported slowly by mammalian P-glycoproteins, but block the binding site for other compounds. However, it has become evident that there are major differences in P-glycoprotein interactions between MOX and the avermectins. All avermectins interfere with P-glycoprotein transport activity with potency similar to IVM. In contrast, MOX is a poor inhibitor of P-glycoprotein-mediated rhodamine123 transport and the EC_50_ for MOX on the ATPase activity of mammalian P-glycoprotein is higher than for the avermectins (Lespine et al., 2007; Ballent et al., 2014).

IVM and MOX display differences in toxicity in several host species, which may be traced, at least in part, to interactions with P-glycoproteins. Entrance of MLs into the brain is restricted by a P-glycoprotein efflux transporter. Ménez et al. (2012) and Janko and Geyer (2013) independently compared the neurotoxicity of IVM and MOX in Mdr1ab(-2/-2) mice, which are P-glycoprotein-deficient. Survival was evaluated over 14 days after subcutaneous administration of each drug (Ménez et al., 2012). LD_50_ values were 0.46 and 2.3 mmol/kg, respectively, for IVM and MOX in these experiments, a 5-fold safety margin (on a dose basis) in favour of MOX. Consistent with this difference, MOX had a lower brain-to-plasma concentration ratio and entered into the brain more slowly than IVM. Brain concentrations determined after administration of LD_50_ doses of each drug were 170–215 and 270 pmol/g for IVM and 830 and 740–1380 pmol/g for MOX in Mdr1ab(-2/-2) and wild-type mice, respectively, indicating that approximately 5-fold higher brain concentrations are required for MOX than IVM toxicity (Ménez et al., 2012). Janko and Geyer (2013) administered oral doses of 0.2 mg/kg to wild-type and the P-glycoprotein mutant mice; brain concentrations of IVM were 67.4-fold higher and those of MOX were 15.6-fold higher in the P-glycoprotein-deficient mice compared with wild-type mice. These authors found a 2.7-fold difference in the toxic dose of MOX compared to IVM, similar to the ratio reported by Ménez et al. (2012); differences in the extent of accumulation of IVM and MOX in the two studies are probably due to pharmacokinetic differences associated with the route of administration.

Differences in the role of ABC transporters, pharmacokinetic profile and neurotoxicity between the avermectins and MOX are important when considering the use of MLs at elevated dose rates and suggest that high doses of MOX will be safer and likely more effective than using higher doses of avermectins in an effort to control ML-resistant parasitic nematodes.

### 7.2 Parasite ABC transporters

The *C. elegans* P-glycoprotein-1 (Cel-Pgp-1) crystal structure has been determined (Jin et al., 2012). Based on this structure, David et al. (2016) developed an *in silico* model of this nematode ABC transporter and estimated the binding energy of IVM, abamectin, eprinomectin, doramectin, selamectin and MOX. The avermectins bound with estimated best fits at between −12 and −13 kcal/mol, i.e., with very high affinity. In contrast, the estimated binding energy of MOX was −10.1 to −10.5 kcal/mol, 2 to 3 orders of magnitude lower affinity. This work showed that the sugar groups on the avermectins played an important role in the high affinity binding of the avermectins for the ABC transporter.

A 3-D model of *H. contortus* P-glycoprotein-13 (Hco-Pgp-13) has been constructed by homology modeling with the Cel-Pgp-1 crystal structure (David et al., 2018). Based on this homology model, the binding energy of IVM was calculated to be −12.8 to −11.2 kcal/mol, indicating high affinity, close to that calculated for Cel-Pgp-1. Hco-Pgp-13 was expressed in recombinant *Pichia pastoris* and the actinomycin A- and IVM-modulated ATPase activity of the parasite P-glycoprotein determined *in vitro*. Immunolocalization studies on *H. contortus* showed that Hco-Pgp-13 was expressed in epithelial, pharyngeal and neuronal tissues, and the authors concluded that the ABC transporter is likely to modulate IVM concentration in the parasite and is likely to play a role in IVM resistance.

In other studies, *H. contortus* P-glycoprotein-2 (Hco-Pgp-2) was found to be expressed in the nematode pharynx (Godoy et al., 2015a). The effect of MLs on rhodamine 123 transport was determined in mammalian cells transfected with Hco-Pgp-2, and IVM and abamectin inhibited rhodamine transport in a saturable manner, with IC_50_ values of approximately 30 nM and 80 nM, respectively. In contrast, the effect of MOX was not saturable (thus an IC_50_ could not be calculated), and MOX inhibition of rhodamine efflux was markedly less than for IVM or abamectin. These data indicate that Hco-Pgp-2 may play a greater role in modulating the concentration of avermectins compared with MOX in critical tissues in the parasite. Given that Hco-Pgp-2 is upregulated in avermectin-resistant worms (Xu et al., 1998), it may play a much greater role in reducing the efficacy of the avermectins compared with MOX.

*H. contortus* P-glycoprotein-16 (Hco-Pgp-16) was also expressed in mammalian cells (Godoy et al., 2015b). Abamectin and IVM markedly inhibited the transport of rhodamine 123 by Hco-PGP-16; in contrast, MOX showed less inhibition of transport by Hco-PGP-16, and the inhibition was not saturable. Similarly, *H. contortus* P-glycoprotein-9.1 (Hco-Pgp-9.1) was also expressed in transfected mammalian cells (Godoy et al., 2016). Again, IVM and abamectin, but not MOX, had pronounced inhibitory effects on the ability of Hco-PGP-9.1 to transport rhodamine 123. These authors concluded that the difference in the interaction of the avermectins and MOX with Hco-Pgp-9.1 and Hco-PGP-16 may help explain the slower rate of development of resistance to MOX compared with the avermectins in *H. contortus*.

P-glycoprotein-9 has also been studied in the equine parasite *Cylicocyclus elongate*. *C. elongate* Pgp-9 was expressed in *Saccharomyces cerevisiae* (AD1-7CegPgp-9V5His cells), and the effect of the fungicide, and P-glycoprotein substrate, ketoconazole on the growth of the yeast was determined. In the absence of ketoconazole, high concentrations of IVM and eprinomectin reduced growth of transfected *S. cerevisiae*, whereas MOX had no effect (Kaschny et al., 2015).

A number of studies point to a role for ABC transporters in reduced efficacy in ML-resistant parasites. Co-administration of a variety of inhibitors of ABC transporters with MOX significantly enhanced sensitivity to this ML in wild-type *B. malayi* females and microfilariae (Stitt et al., 2011). Resistant *Cooperia oncophora* worms surviving exposure to IVM and MOX were able to induce P-glycoprotein-11 transcription (De Graef et al., 2013), an effect not observed in susceptible worms. A *Parascaris equorum* homolog of Pgp-11 was upregulated in an ML-resistant strain and was shown to be expressed in the gut (Janssen et al., 2013a). Expression of this protein in a Pgp-11 null mutant strain of *C. elegans* caused diminished sensitivity to ivermectin (Janssen et al., 2015). The phenotype of ML-resistance in *C. oncophora* larvae was reversed by exposure to the P-glycoprotein inhibitor verapamil (Demeler et al., 2013a) and adult stages of this strain showed a significant 2-fold increase in expression of P-glycoprotein-12 (Con-Pgp-12) in response to MOX, but not to IVM exposure (De Graef et al., 2013). P-glycoprotein-2 levels were approximately 3 times higher in IVM/abamectin-resistant *H. contortus* than in susceptible worms, and IVM treatment increased P-gp-2 levels by 2-fold at 0.5 day and at 1 day, whereas there was no significant increase in P-gp-2 levels after MOX treatment. These changes in P-gp-2 levels appear to be important for efficacy against the resistant worms, as IVM and abamectin failed to reduce egg counts of the resistant worms, whereas MOX decreased fecal egg count by 86.7% (Lloberas et al., 2012a). Finally, Bygarski et al. (2014) found differences in P-glycoprotein expression induced by MOX and IVM in several strains of *C. elegans*, suggesting differences in the efflux mechanisms between these MLs. Taken together, there is abundant evidence that MOX and avermectins interact differently with nematode P-glycoproteins, and that overexpression of P-glycoproteins in avermectin-resistant nematodes is likely to reduce the potency of avermectins, with less effect on MOX.

## 8 Toxicology

While all MLs share a set of common chemical and pharmacological characteristics, they are also distinguished by important quantitative differences. These differences are evident in parameters related to pharmacokinetics and receptor binding, as well as the potential for toxicity in companion animals, as summarized previously (Geyer and Janko, 2012; Prichard et al., 2012; Merola and Eubig, 2018) and discussed above. The presence of a functional drug efflux pump (MDR1) at the blood-brain interface is essential for limiting brain penetration of MLs, including MOX, in dogs (Geyer and Janko, 2012), and a similar situation appears relevant in cats, in which MDR mutations have been reported in a subset of animals with ML toxicosis (Mealey and Burke, 2015).

Recent research on the safety profile of MLs in mice with loss-of-function mutations in the *mdr1-a* gene, introduced above, confirmed that these animals are more susceptible to ML toxicity than wild-type mice, but with similar symptomatology when toxic doses are reached (Swain et al., 2013). The therapeutic index of MOX in these animals is greater than that of IVM (Ménez et al., 2012; Janko and Geyer, 2013). Independent studies in recombinant mouse strains engineered to express the mutated canine MDR1-A protein in place of the wild-type murine homolog showed that MOX and IVM attained roughly similar concentrations in the CNS following dosing, but that MOX was less intrinsically toxic on a molar basis (3–4 -fold), an observation attributed to the lower affinity of MOX than IVM for mammalian ligand-gated ion channels (Ménez et al., 2012; Janko and Geyer, 2013). Transcriptomic analyses of recombinant mouse brains following exposure to PGP substrates including IVM and MOX revealed a generally similar pattern of changes in genes related to the behavioral effects of ML intoxication, suggesting the presence of some common responses to brain accumulation (Zhu et al., 2014).

Although the primary treatment of companion animals exhibiting signs of CNS ML toxicosis remains symptomatic support until the drug concentration subsides below the toxic level, some evidence suggests that infusions of a lipid-rich nutritional support can speed recovery, presumably by providing a peripheral sink to extract the drug from the CNS compartment (Bates et al., 2013). Additional evidence is required to make this a standard intervention.

Efforts to develop MOX for human use included Phase I-III trials for onchocerciasis (discussed above), which included dose-ranging and safety studies. The dose chosen for phase III efficacy trials, 8 mg orally, was associated with a set of non-severe side effects, all of which subsided without intervention (Awadzi et al., 2014; Opoku et al., 2018). MOX then gained FDA registration in 2018, at this dose, for prolonged reduction in dermal microfilariae in humans infected with *O. volvulus*. Additional safety studies may be required to extend the registration of MOX for human scabies treatment, which is predicted to require higher doses (Mounsey et al., 2016). Encouragingly, no significant side effects were reported following an 8 mg dose of moxidectin in 64 patients treated for infection with *S. stercoralis* (Barda et al., 2017).

## 9 Ecotoxicology

ML endectocides, including MOX, have at least some activity against arthropods as well as nematodes, but vary in spectrum and toxicity to arthropod species in the environment (Lumaret et al., 2012; Prichard et al., 2012). Recent research published on the ecotoxicity of MOX confirms earlier findings and suggests that, despite the more prolonged environmental exposure profile of MOX in feces released from treated animals, the drug generally poses less of a threat to non-target organisms in the environment following excretion in feces into the environment than do other MLs (Jacobs and Scholtz, 2015).

Noteworthy advances in this regard were derived from studies on the sensitivity of multiple species of dung flies to MLs (Blanckenhorn et al., 2013), which found that the toxicity of MLs varies considerably across the phylogenetic range of dung flies, and that sensitivity to MOX, though lower in potency, was correlated with sensitivity to IVM, suggesting that variations in either internal accumulation or target affinity are conserved among these arthropod species. A detailed study of the toxicity of MOX and IVM in juvenile and adult dung beetles (*Scarabaeus cicatricosus*) using multiple physiological and developmental assays found that MOX is 5–6 times less potent than IVM against these organisms (Verdú et al., 2018), a finding consistent with previous work. Based on threshold concentrations required for observable effects, this study also reported that fecal concentrations of MOX declined to sub-toxic levels more quickly than for IVM (2 vs. 4 weeks). However, affirming that species differences can be anticipated in this regard, a study based on the high affinity of MOX for particles in an aqueous environment, tested the toxicity of MOX in cattle feces for several aquatic organisms (Mesa et al., 2018). MOX was less potent than IVM against an amphipod and a zooplankter, but was more toxic than IVM for the snail *Pomacea canaliculata*. Whether other snail species share this pattern of toxicity, and whether effects on snail populations are apparent in water bodies (or other invertebrate species in pastures) that are near intensive cattle operations, are research questions that deserve analysis (Wall and Beynon, 2012).

## 10 Resistance to macrocyclic lactones

### 10.1 The current landscape of macrocyclic lactone resistance in parasitic nematodes

Resistance to ML endectocides has been known for decades (Sangster et al., 2018). Phenotypic resistance has been reported in multiple species of parasitic nematodes that infect almost every host species of veterinary importance; increasing numbers of such reports are available from global sources and are too numerous to discuss here, although recent reviews are valuable in this regard (Molento et al., 2012; von Samson-Himmelstjerna, 2012; Leathwick and Besier, 2014; Matthews, 2014; Geurden et al., 2015; Wolstenholme et al., 2015; Bellaw et al., 2018). Anthelmintic-resistant parasites continue to pose global challenges for control and impose significant health and economic costs on animals and their owners, especially livestock producers.

#### 10.1.1 Resistance in gastrointestinal parasites

Although it is common to consider all MLs as a class, it is clear as illustrated above that members of the group can be distinguished (Prichard et al., 2012), even if the pharmacological bases for these differences remain incompletely understood. In this regard, differences in the extent of resistance to MOX compared to IVM and other avermectins in GI nematodes continue to be apparent. Of note in this regard are recent *in vitro* and *in vivo* studies on the pharmacology of ML resistance in these parasites.

Multiple *in vivo* studies confirm that standard doses of MOX remain at least somewhat efficacious against parasite strains that are resistant to IVM and other MLs, although it is important to stress that resistance to MOX is present in GI species and can lead to treatment failure. Notable (not exhaustive) examples of the relative efficacy of MOX in these situations include *H. contortus* in lambs (Lloberas et al., 2015), *O. ostertagi* in cattle in New Zealand (Waghorn et al., 2016), *Haemonchus* and *Cooperia* spp. in feedlot calves in Argentina (Fazzio et al., 2016), *C. onchophora* in cattle in Scotland (Bartley et al., 2012) and trichostrongyloid nematodes in Brazil (Lopes et al., 2014). In these field studies, although MOX performed better than avermectins, a level of resistance to MOX (e.g., efficacies between 80 and 90%) was nevertheless evident. However, these reduced, but still moderately high efficacies can still be useful in preventing production losses from parasitism, but this utility can be lost by misuse/overuse of the anthelmintic. Few studies have evaluated whether the quantitative extent of resistance is the same for all MLs in this context by conducting comparative dose-response trials in infected animals, although some notable work has been done in this area. Comparisons of the pharmacodynamics of IVM administered to lambs infected with IVM-resistant strains of *H. contortus* revealed that a 5-10-fold increase in the dose of IVM was highly effective (but not a 2-fold increase), suggesting that the resistance ratio for this drug against adult stages is 5–10 (similar to what is observed in larval development assays; see below) (Alvarez et al., 2015; Lloberas et al., 2015).

Results from recent *in vitro* studies with larval stages of trichostrongyloid nematodes have confirmed differences in relative resistance among the MLs. Resistance to IVM, but not to MOX, was detected in larval *Cooperia spp*. in isolates obtained from Brazilian cattle (Almeida et al., 2013). ML-resistant and sensitive isolates of *H. contortus* and *H. placei* were compared for sensitivity to commercially available MLs in a larval development assay (Kotze et al., 2014b). Although resistance ratios were modest and similar among MLs for an IVM-resistant isolate of *H. placei* (1.7–3.3-fold), clear differences in the extent of resistance were found for *H. contortus*, ranging from 4-fold for MOX to 13-fold for IVM to 70-fold for eprinomectin. A similar study used a larval development assay to test resistance to multiple MLs in *H. contortus, Trichostrongylus colubriformis* and *O. ostertagi* (Demeler et al., 2013b); these authors also found significant differences in resistance ratios among the three species and the various MLs tested, with MOX consistently displaying the lowest extent of resistance. Interestingly, direct measurements of ML effects on migration of L3 stages of these parasites revealed a different pattern of relative resistance, with generally lower resistance ratios; in these assays, MOX had the highest resistance ratios, although very high drug concentrations were needed to see any effect. Subsequent studies using larval stages of *H. contortus* and *C. oncophora* led to the conclusion that assays of larval motility and migration were poorly suited for characterizing ML resistance (George et al., 2018).

The bases for differences in this regard between the two species and among the MLs remain incompletely resolved, but point to the challenges of finding simple, broadly applicable causes of ML resistance in these parasites. In addition, the relevance of drug resistance in larval stages vs. adults is under-investigated for trichostrongyloid species. In that regard, a study using adult stages of *H. contortus* and *Ostertagia circumcincta* offered new insights into the phenotype (Demeler et al., 2014). Various MLs were exceptionally potent in directly affecting muscle contractions in adult *H. contortus*, and resistance ratios were orders of magnitude greater than observed in larval development assays (>11,000-fold for IVM), but again varied among MLs (lower for milbemycins compared with avermectins). Drug effects on motility of intact adult *O. circumcincta* showed lower resistance ratios than the muscle contractility assay, although still much greater than in larval assays (600-fold for IVM); resistance to MOX was the lowest among the MLs tested. These data suggest that the extent and mechanistic basis for ML resistance may differ between adults and larvae, but this conclusion clearly requires confirmation and extension to additional species and strains, and is not necessarily compatible with the limited amount of available data from dose-response assays in infected sheep (see above).

#### 10.1.2 Resistance in heartworm (*Dirofilaria immitis*)

Phenotypic resistance to ML preventatives is present in heartworm populations in North America (Pulaski et al., 2014; Bourguinat et al., 2015; Blagburn et al., 2016; McTier et al., 2017a; Ballesteros et al., 2018). Genotypic associations have been investigated for this phenotype and it is apparent that loss-of-efficacy and proven resistant isolates share a set of single nucleotide polymorphisms (SNPs) that serve as molecular markers for the trait (Bourguinat et al., 2015). Breakthrough infections have been reported in controlled laboratory conditions for every approved ML, although comprehensive evaluations of the quantitative extent of resistance and experiments to determine variations in the phenotype for different strains or for different MLs are not available. *In vitro* assays measuring ML effects on larval migration or microfilarial motility have not proven informative for this phenotype (Evans et al., 2017; Maclean et al., 2017), supporting the hypothesis that the effects of IVM (and presumably other MLs) are caused by alteration of the host-parasite interface (Geary and Moreno, 2012). However, a surrogate *in vivo* assay measuring the effect of ML treatment on microfilarial burden in the peripheral circulation has been proposed as an alternative diagnostic test for ML resistance (Geary et al., 2011; Moorhead et al., 2017) based on the presumption that the mechanism of resistance is conserved in all larval stages.

In this context, experience with an ML-resistant isolate is informative (Bourguinat et al., 2011). Persistent microfilaremia in a dog following adulticidal treatment was unaffected by high doses of IVM (0.2 mg/kg) and milbemycin oxime, including 7 and then 8 consecutive days of dosing with milbemycin oxime at 2 mg/kg. These data suggest that the strain in question (which carried the SNP markers associated with ML resistance) was highly resistant to IVM and milbemycin oxime. Intriguingly, microfilaria of the ZoeMO isolate, a relative of the highly ML-resistant JYD-34 isolate which also carries the SNP resistance markers, were essentially eliminated over several months in dogs treated with MOX extended release products, but were incompletely reduced by a single high oral dose of moxidectin (0.25 mg/kg) (McTier et al., 2017b).

Although a single oral dose of 3 μg/kg MOX was ineffective at protecting dogs from infection with JYD-34 and other ML-resistant strains (McTier et al., 2017a), a single topical dose (2.8–6.7 mg/kg) of MOX was fully effective in preventing infection with JYD-34 in treated dogs, while 3 consecutive monthly doses of IVM, selamectin and milbemycin oxime were not (Blagburn et al., 2016). The topical MOX formulation provides exceptionally prolonged exposure, unlike the other MLs, with high plasma levels present more than a month after dosing (Bowman et al., 2016). Coupled with the microfilaricidal data obtained with extended-release MOX products, these results suggest that prolonged exposure to MOX (and perhaps other MLs) may be efficacious against otherwise ML-resistant strains of *D. immitis*, a possibility that demands further experimental analysis. However, it is important to note that the quantitative extent of resistance may not be the same for all MLs or for all heartworm isolates, and that exposure profiles may alter efficacy against at least some ML-resistant strains of *D. immitis* (Blagburn et al., 2016). The concept that prolonged exposure can enhance efficacy against resistant parasites is supported by data showing that heartworm infection at the end of the protective period associated with ProHeart^®^ 6 treatment was ineffective at preventing infection with a ML-resistant strain (Bourguinat et al., 2015), while infection with a resistant strain 2 days after a standard ProHeart^®^ 6 dose was almost completely prevented (1 male worm present in 6 treated dogs) (Bowman et al., 2017).

The microfilarial reduction test (Geary et al., 2011) was employed in a case-controlled trial involving veterinary clinics across the USA to determine the correlation between a set of SNPs consistently found in ML-resistant isolates and the efficacy of a single microfilaricidal dose of topical MOX (as Advantage Multi^®^; Bowman et al., 2015) (Ballesteros et al., 2018). Samples of microfilariae were taken from volunteer dogs for analysis of molecular markers. The dogs were then treated with topical MOX. The dogs returned to the clinic for a second microfilarial sample for genotyping and the reduction in microfilaremia was determined. Responses to treatment ranged from complete or almost complete removal of microfilariae to a moderate response (roughly 50% reduction) to no effect on microfilaremia. There was a strong association between the SNPs and the extent of microfilaricidal efficacy; a combination of two SNPs was highly predictive of the therapeutic response. Interestingly, surviving microfilariae recovered from dogs that showed moderate efficacy responses (50% reduction in microfilarial load) showed no change in SNP allele frequency compared with the initial microfilarial samples, suggesting the presence of parasite populations with an intermediate degree of resistance rather than a mixture of fully resistant and fully sensitive populations. This observation is also consistent with efficacy results obtained for a ML-resistant isolate of *D. immitis* sampled independently many months apart, in which distinct subsets of microfilariae from the same infected dog were used to develop pools of L3-stage parasites for infection of naïve dogs (see Vidyashankar et al., 2017). In these dogs, the efficacy of a single dose of MOX as a preventative was low, but varied significantly between the two sampled parasite populations. Although many possible explanations for this observation are possible, it may reflect the presence of parasite populations with varying degrees of resistance due to ongoing production of microfilariae that inherit various combinations of alleles. More work is clearly needed to understand these interesting observations that could have important implications for managing resistance in heartworms. Interestingly, the same authors found that multiple doses of MOX were more than twice as effective in reducing infection compared with single doses, again consistent with the concept that the duration of exposure to MOX is an important determinant of efficacy against ML-resistant parasites (Vidyashankar et al., 2017).

### 10.2 Mechanisms of resistance, selection and relative potency of macrocyclic lactones

Despite considerable investment in research on the molecular mechanisms of resistance to ML endectocides, our understanding of this situation remains imprecise (Kotze et al., 2014a; Whittaker et al., 2017). Too few studies have systematically and quantitatively compared different anthelmintic resistant strains of a single species or resistant strains of multiple species vis-à-vis changes in sensitivity to various MLs in larvae and adults. It therefore remains unclear if mechanisms of resistance are shared to the same extent or even identical in different developmental stages of parasitic species, or across different species and for different drugs. Available data strongly indicate that ML resistance is a multigenic trait, but no “smoking gun” individual gene has reliably been found to account for resistance. Major limitations to fully defining the genotype-phenotype connection in ML resistance include the very complex genomics of trichostrongyloid species and the lack of convenient and robust small laboratory animal models that can economically support the full life cycle of important species of veterinary relevance.

Recent research in this area has highlighted the possible contribution of P-glycoprotein -mediated drug efflux systems to the ML resistance phenotype, as noted above. For some stages and some species, some efflux inhibitors can markedly reverse ML resistance while also increasing sensitivity to these drugs in wild-type strains (Demeler et al., 2013a; AlGusbi et al., 2014; Heckler et al., 2014; Raza et al., 2015). However, these effects are not uniform in extent, vary with species and inhibitor and have focused mostly on IVM.

Recent studies on ML resistance in the free-living nematode *C. elegans* highlighted possible roles for P-glycoproteins and drug detoxification mechanisms in this phenotype (Ardelli and Prichard, 2013; Janssen et al., 2013b; Bygarski et al., 2014; Ménez et al., 2016, 2019), supporting conclusions drawn from experiments using inhibitors to modulate IVM sensitivity in larval stages of parasitic species. However, it is not possible to draw simple or universal conclusions even from experiments on this model organism. Selection of a highly resistant strain of *C. elegans* by sequential, increasing exposure to IVM caused profound upregulation of two P-glycoproteins, and RNAi suppression of one of these (Pgp-12) reversed the phenotype of IVM resistance (Figueiredo et al., 2018). These results differ from those obtained in a previous study on a different IVM-selected strain of *C. elegans*, in which the expression of other P-glycoproteins was elevated, but no individual gene in this family could account for resistance to IVM (Yan et al., 2012).

Comparisons of MOX and IVM in some of these strains revealed cross-resistance but with significant differences; the extent of resistance was significantly greater to IVM than to MOX, as is the case in parasitic species (Ménez et al., 2016). Resistant strains were also characterized by alterations in the structure and presumably function of amphidial neurons in *C. elegans*, a phenotype long associated with ML resistance in this organism, and also seen in *H. contortus* (Urdaneta-Marquez et al., 2014). These findings support the concept that ML resistance is polygenic in nematodes, may be acquired in a step-wise fashion (Ménez et al., 2019) and that the MLs are differentially affected by the particular combination of genes involved.

If drug efflux mechanisms underlie at least part of the phenotype of ML resistance, then resistant strains should accumulate lower concentrations of these drugs at equilibrium, or be shown to expel them more readily after exposure *in vitro* or *in vivo*. Few relevant experiments have been reported, but some evidence suggests that caution is needed before accepting the conclusion that P-glycoprotein mechanisms are primary drivers of ML resistance in adult stages of parasitic nematodes; although they may play an essential early role in a step-wise process of resistance development. For example, exposure of ML-resistant adult *H. contortus in vivo* to various doses of IVM altered P-glycoprotein expression to only a minor degree (Lloberas et al., 2012a; Alvarez et al., 2015; Maté et al., 2018), and doses of MOX had no effect on this parameter; it is not clear that the changes observed after IVM exposure were of sufficient magnitude to account for the resistance phenotype (Maté et al., 2018). Furthermore, direct measurements of the concentration of IVM and MOX in ML-resistant adult *H. contortus* recovered from treated sheep revealed that the concentrations associated with the parasites were similar to those in the surrounding abomasal material, suggesting that an equilibrium distribution had been established between the parasite and its environment (Lloberas et al., 2012b, 2015; Lifschitz et al., 2017). It would be extremely informative to perform similar studies with a sensitive strain of this parasite *ex vivo* to compare with data from the resistant strain (Lloberas et al., 2015). In this context, it is important to reiterate that ML resistance may be acquired in a step-wise fashion, with initial mechanisms perhaps associated with changes in expression of ABC transporters (Ménez et al., 2019) and the interaction of different MLs with those transporters, being supplemented or even replaced, following continued selection, by mechanisms having stronger effects on the extent of resistance.

Finally, although genomic analyses have not yet been able to conclusively identify resistance genes in either GI parasites or heartworms, research done on backcrosses of ML-sensitive and -resistant *H. contortus* have found a strong association with a genomic locus defined by microsatellite Hcms8a20, further refined to a specific QTL on chromosome 5 in two independent crosses (Rezansoff et al., 2016; Doyle et al., 2019). Candidate gene loci proposed to underlie this phenotype, including some that have previously been associated with ML resistance in multiple *H. contortus* isolates (e.g., *dyf-7*; Urdaneta-Marquez et al., 2014) and selected P-glycoprotein genes, were not associated with the ML resistance trait in these crosses; the authors conclude that this QTL may contain a novel gene that is the primary factor underlying IVM resistance in these crossed populations. Interestingly, similar work on a cross of a multi-drug resistant isolate of *Teladorsagia circumcincta* suggested that a P-glycoprotein locus is associated with IVM resistance in this parasite (Choi et al., 2017). Clearly, additional work is needed to illuminate the genes associated with IVM resistance in these trichostrongyloid species, and to determine to what extent sensitivity to other MLs is altered in them.

It is important to consider whether ML exposure may select for different resistance mechanisms in different life stages of trichostrongyloid parasites. Free-living larval stages in the environment are exposed to selection pressure from microbially-derived xenobiotics and may be able to express protection/detoxification strategies to limit xenobiotic toxicity, much like the free-living species *C. elegans* (Burns et al., 2010). In contrast, the adult worms residing in a mammalian host, which is a parasitic stage confronted by drug selection following treatment, are protected to some degree by host detoxification mechanisms and may not as readily turn on endogenous detoxification/protection pathways as can larvae that must develop and survive in feces contaminated with MLs, instead undergoing selection for different resistance mechanisms. More research is urgently needed in this area to illuminate novel strategies in order to rationally counteract ML resistance in adult stages of GI nematodes.

## 11 Attributes of moxidectin that may reduce selection for resistance and/or enable control of ML-resistant parasites

Because the quantitative extent of ML resistance in target-stage populations of parasitic nematodes has not been intensively investigated and experiments on the selection of AR strains in species of veterinary importance remains expensive and challenging, the possible influence of pharmacodynamic manipulations on control of AR strains have been insufficiently investigated, although promising developments have been reported.

It has been known for decades that extending the intensity or duration of exposure of anthelmintics in treated animals enhances efficacy, even against otherwise AR strains. This phenomenon has been shown for benzimidazoles and IVM for trichostrongyloid nematodes (Le Jambre et al., 1981; Ali and Hennessy, 1996; Hennessy, 1997; Barrère et al., 2012). It is also clearly evident in the macrofilaricidal action of flubendazole, for which prolonged exposure to low concentrations *in vivo* is much more effective than shorter exposures to high concentrations (Mackenzie and Geary, 2011; Geary et al., 2019). These findings form the basis of a more rational choice of dosing regimens to enhance efficacy of MLs, including MOX, and other anthelmintics (Lanusse and Prichard, 1993; Lloberas et al., 2012b; Lanusse et al., 2014, 2018, 2016; Leathwick and Luo, 2017; Lifschitz et al., 2017; Fazzio et al., 2019) based on increasing either the intensity of exposure to the anthelmintic at the site of infection, or the duration of exposure, although neither manipulation is uniformly able to fully overcome AR and may vary with different MLs.

With specific regard to MOX, similar results have been obtained for routes of administration that lead to different exposure profiles and thus different efficacy in cattle (Leathwick and Miller, 2013; Fazzio et al., 2019), and higher doses delivered to the site of infection are effective against ML-resistant parasites in sheep (Lloberas et al., 2015). It is important to consider the exposure profile as a composite of both maximum concentration and duration of exposure to effective concentrations. MOX is a highly potent anthelmintic and ML endectocide. However, its relative potency compared with the avermectins may vary depending on the target parasite (e.g., endoparasites versus ectoparasites), life-cycle stage (adult or larval) and potency assay (*in vivo* versus *in vitro*, etc.). However, it is important to recognize that MOX seems to be the most potent ML against susceptible filaria, including heartworms (McTier et al., 1992) and *O. volvulus* (Opoku et al., 2018). Altering the exposure profile in terms of dose rate and duration of exposure offer intriguing possibilities to extend the therapeutic utility of ML endectocides in veterinary practice.

Cross- (or side-) resistance between MOX and other MLs is evident in many parasite species (and in *C. elegans*), although the extent of resistance is typically significantly lower. Continued exposure to MOX in the field can lead to therapeutic failure at label doses (Condi et al., 2009; Gasbarre et al., 2009; El-Abdellati et al., 2010; Paraud et al., 2016; Canton et al., 2017; Ploeger and Everts, 2018; Nagata et al., 2019); whether further prolonging drug exposure in animals infected with highly resistant strains could maintain adequate control over time should be experimentally evaluated.

Lastly, it is increasingly urgent to find new anthelmintics to augment the available pharmacopeia for veterinary (and human) therapy. In this regard, combining a new mode of action product with an existing anthelmintic which retains good activity has been advanced as a possible strategy to retard the development of resistance. Based on what is known about the pharmacodynamics/pharmacokinetic properties and resistance profile of MOX, it is likely to be an opportune candidate for combination with new anthelmintics, once they are developed.

## 12 Perspectives and conclusions

ML anthelmintics have enjoyed a unique position in the therapeutic arsenal against parasitic nematodes and account for approximately 80% of the veterinary anthelmintic market. In addition, IVM has been vital to filarial control programs in humans, and MOX has recently been registered for human use. However, these ‘wonder drugs’ (Geary, 2005) are seriously threatened by the development of resistance to them. Resistance developed initially in small ruminants to the avermectins and subsequently to MOX. As reviewed here and elsewhere, there is good evidence that MOX selects less rapidly for resistance than the avermectins and remains more potent than the avermectins against nematodes exhibiting ML resistance (Prichard et al., 2012; Ménez et al., 2016). There are many examples in the literature of the continuing efficacy of MOX for parasite control despite evident resistance to the avermectins (Blagburn et al., 2011; Prichard et al., 2012) (although it is clear that MOX-resistant parasite populations have evolved).

The mechanism(s) of ML resistance remains to be proven, and to what extent ML resistance is a polygenic vs. monogenic trait in various species of parasitic nematodes has yet to be resolved. Available evidence suggests that ABC transporters may play a role in this phenotype, as discussed above. In this context, it is important to note that the avermectins are better substrates for ABC transporters than MOX, a characteristic that may contribute the initial step in the development of resistance to the avermectins (Ménez et al., 2019). ABC transporters may reduce the concentration of an ML reaching receptors in the parasite, and ABC transport inhibitors may block the efflux of MLs from nematodes and increase ML efficacy (Lespine et al., 2012).

However, the contribution of ABC transporters to the phenotype of ML resistance across members of the phylum Nematoda and in different life-cycle stages of these parasites remains incompletely characterized. More research, including definitive studies on the uptake, accumulation and efflux of IVM and other MLs from wild-type and ML-resistant parasites, is urgently needed to clarify this situation. More work to define the genomics of ML resistance in field-derived populations of parasitic nematodes (Rezansoff et al., 2016; Choi et al., 2017; Ballesteros et al., 2018; Doyle et al., 2019) should also be of the highest priority, with the goal of identifying the major genes that contribute to the phenotype. Quantitative studies of the extent and variation in ML resistance among members of this class of drugs in different species and populations of parasitic nematodes are also badly needed. Our limited understanding of the basis for ML resistance hampers our ability to devise appropriate therapeutic strategies to provide veterinarians and animal owners/producers workable solutions to overcome current challenges to parasite control.

In this regard, it is imperative to consider the role of potency, dose and pharmacokinetic properties of a particular ML in achieving efficacy against parasites in the face of developing ML resistance. A review of available data suggests that the high fat solubility, long half-life, intrinsic potency and high safety margin of MOX could allow this drug to be used at sufficiently high doses to safely remove avermectin-resistant parasites (see Box 1). We have reviewed data that suggest that extended duration of exposure to MLs, particularly MOX, may be beneficial for removal of ML-resistant GI parasites and heartworms. This possibility is in urgent need of systematic experimental testing. Work is needed to determine if duration of exposure is the most important variable for this effect, or if other pharmacological properties of MOX significantly contribute to it.

Box 1 **Characteristics of MOX that can be exploited to improve control of ML-resistant parasites**

1. Relatively high potency against ML-resistant nematodes
2. Unique structural characteristics which help distinguish MOX from avermectins on GluCl receptors and ABC transporters
3. Pharmacokinetic profile
  a. High lipophilicity (logP 5.4)
  b. Limited biotransformation
  c. Low interaction with mammalian and parasite ABC transporters
  d. Long half-life
4. High target host safety
a. Lower risk of mammalian neurotoxicity compared with avermectins such as ivermectin
5. Low ecotoxicity
6. High flexibility in terms of route of administration and formulation
7. Good profile for use in combination anthelmintic products
8. High suitability for high dose, high potency, long-acting formulations

Alt-text: Box 1

The analysis of available data suggests that there may be an opportunity to delay ML resistance selection and maintain high efficacy against parasites that are resistant to other MLs at currently used doses. Whether this strategy will be beneficial in the long-term requires further research to develop new formulations of MOX that safely and conveniently deliver high doses to provide reliable and sustained efficacy in the field. However, given the significant challenges to finding a new class of anthelmintics that matches the breadth of spectrum, potency and safety of the MLs, we emphasize that the unique properties of MOX justify further research on this molecule to develop better ways to control parasites in the face of developing anthelmintic resistance. If we are to prolong the usefulness of MOX as a critical tool for parasite control, it should be used intelligently, exploiting its remarkable pharmacological potential to realize these benefits without imposing selection pressure for high level drug resistance. Key questions for research that address some of the most important outstanding questions around a better understanding of how MOX or other MLs may be better used to maintain control of nematode parasites in the face of developing resistance are summarized in Box 2.

Box 2 **Outstanding research questions and challenges**

1. Modeling the interaction of MOX and other MLs with GluCl receptors, including estimating binding affinities.
2. Mapping where different GluCls are expressed in different parasitic nematodes, including filaria.
3. Characterising the interaction of MOX and other MLs with parasite ABC transporters, including transport studies and molecular modeling.
4. Determining the mechanisms and genetics of ML resistance, and how these mechanisms differentially affect different MLs.
5. Defining the role of structural changes in sensory neurons in ML resistance in different parasites.
6. Comparing the genetics of ML resistance in different isolates of the same parasite species and in different nematode species.
7. Defining the relative role of different mechanisms of ML resistance in different life-cycle stages of parasites.
8. Ex vivo studies comparing anthelmintic uptake, accumulation and efflux in tissues of susceptible and resistant strains of nematodes.
9. Identifying the fitness cost of ML resistance in different parasites, and how this can be exploited to sustain the effectiveness of MLs.
10. Rapid, sensitive and predictive *in vitro* methods to detect and quantify ML resistance in heartworm and other parasites.
11. Evaluation of ecotoxicity from high dose rate, long-acting MOX formulations.
12. Determining the efficacy of high dose rate MOX, and other MLs, against strains of parasites that are resistant to currently used dose rates.
13. Establishing whether resistance to ML heartworm preventives can be overcome with repeated monthly treatment or higher dose rates of MLs, keeping in mind the need to retain target host safety.
14. Defining the role of duration of exposure on potency against ML-resistant parasites.
15. Determining the impact of currently recommended parasite control strategies on the selection and spread of ML-resistance.
16. Research on rational combinations of new molecules with existing molecules.

Alt-text: Box 2
